# Assessing the negative impact of phenyl alkanoic acid derivative, a frequently prescribed drug for the suppression of pain and inflammation, on the differentiation and proliferation of chondrocytes

**DOI:** 10.1186/s13018-016-0406-x

**Published:** 2016-06-30

**Authors:** Seyit Ali Gumustas, İbrahim Yilmaz, Mehmet Isyar, Duygu Yasar Sirin, Ahmet Guray Batmaz, Ali Akin Ugras, Kadir Oznam, Zafer Ciftci, Mahir Mahirogullari

**Affiliations:** Department of General Secretariat of the Public Hospitals Union, Republic of Turkey, Ministry of Health, 59100 Tekirdag, Turkey; Department of Pharmacovigilance, Materiovigilance and Rational Use of Drugs, Republic of Turkey, Ministry of Health, 59100 Tekirdag, Turkey; Clinics of Orthopaedic and Traumatology, Central Hospital Health Group, 34742 Istanbul, Turkey; Department of Molecular Biology and Genetic, Namik Kemal University Faculty of Arts and Sciences, 59100 Tekirdag, Turkey; Department of Orthopaedic and Traumatology, Istanbul Medipol University School of Medicine, 34214 Istanbul, Turkey; Department of Otolaryngology and Head and Neck Surgery, Namik Kemal University School of Medicine, 59100 Tekirdag, Turkey; Department of Orthopaedic and Traumatology, Memorial Hospital, 34384 Istanbul, Turkey

**Keywords:** Phenyl alkanoic acid, Chondrotoxicity, Proliferation, Stage-specific, Embryonic, Antigen-1

## Abstract

**Background:**

Non-steroidal anti-inflammatory drugs (NSAIDs) are frequently prescribed to relieve pain and inflammation. These NSAIDs have also analgesic effects and can be administered via oral, injectable, and topical routes. During inflammation, a number of synovial mediators and cytokines are released which decrease the pH level of the synovial fluid. Administration of acidic NSAIDs further decreases the pH levels and hence contributes to the destruction of the cartilage. To our knowledge, no cellular-based study regarding the chondrotoxicity of phenyl alkanoic acid derivatives on NSAIDs was conducted before. Thus, the aim of this pioneering study was to examine the effect of frequently prescribed NSAIDs, a phenyl alkanoic acid derivative, flurbiprofen, on the proliferation and differentiation of human primer chondrocyte cultures in vitro.

**Methods:**

Primer chondrocyte cultures were prepared from osteochondral tissue obtained during surgery for gonarthrosis. Samples not exposed to the pharmacological agent were used as the control group. The samples were treated with 1, 10, 100, 250, 500, or 1000 μM of the agent for 24, 48, and 72 h. The cell viability, toxicity, and proliferation were assessed with MTT (3-(4,5-dimethylthiazol-2-yl)-2,5-diphenyltetrazolium bromide) analysis and prechondrocytic precursor stage-specific embryonic antigen-1 (SSEA-1) expression using a commercial ELISA kit spectrophotometrically. The surface morphology of the samples in each group was compared using an inverted light microscope and an environmental scanning electron microscope (ESEM). An analysis of variance was used to compare between-group differences. Tukey’s honest significant difference (HSD) method (95 % confidence interval) was used to evaluate the differences and significance in averages. The alpha significance value was considered <0.01.

**Results:**

Statistically significant cytotoxicity was observed in the treatment groups. NSAID had a significant negative effect on the proliferation and differentiation of chondrocytes as compared to the control group (*p* < 0.01).

**Conclusion:**

Before administering phenyl alkanoic acid derivatives in the clinical setting, their role in suppressing the proliferation and differentiation of chondrocytes should be taken into account. Thus, caution should be given when prescribing these drugs.

## Background

During cartilage production and degradation cycles, equilibrium exists between the amount of catabolic cytokines like interleukin-I, tumor necrosis factor alpha, nitric oxide, matrix metallopreoteinases, and their inhibitors. Hence, it can be possible to establish a balance between the levels of collagen types I and II, which are responsible for the increased fibrocartilage and hyaline cartilage production, respectively [1–5]. Moreover, in order to maintain this equilibrium, the pH values of the environment that the cartilage tissue resides should be within the alkaline range. In case the pH values decrease and the cartilage tissue is surrounded by a rather acidic milieu, a degeneration process is inevitable and overt signs of cartilage toxicity may be witnessed [6, 7].

During the course of the chronic disorders including rheumatoid arthritis, osteoarthritis, ankylosing spondylitis, tendinitis, bursitis, gout, and juvenile chronic arthritis, a number of substances are released into the medium following tissue damage and a concomitant decrease in the activity of the inhibitor mechanisms is observed. These conditions are characterized by significant inflammation and increasing levels of pain in the affected regions of the body. The inflammation has also the propensity to decrease the pH levels and render the milieu more acidic. Furthermore, the drugs employed to relieve pain and inflammation, specifically non-steroidal anti-inflammatory drugs (NSAIDs), are known to decrease the pH levels to more acidic values. As a consequence, pain, cartilage tissue damage, and functional deficits become inevitable [8].

A similar problem is also experienced following sino-nasal or septal surgeries. Irrespective of the method utilized, either conventional septoplasty, radiofrequency, or LASER, a significant and unpredictable amount of cartilage injury was reported to occur [9, 10]. Following these surgeries, the patients usually receive NSAID’s for the relief of pain and the synergistic effect of these negative factors are usually being overlooked.

To suppress inflammation and pain, a number of non-steroidal anti-inflammatory drugs, which also have analgesic activities, are usually prescribed [8]. However, majority of these drugs are composed of organic acids and within the circulation they display great affinity for binding to the plasma proteins. It is well known that, following an inflammation process, the pH values become acidic and the tissue becomes more permeable to the plasma proteins. These factors enable accumulation of the plasma protein-bound NSAID’s in the inflamed region. The decreased pH levels also increase the amount of the non-ionized lipid soluble portion of the drug, which in turn increase the interaction between the lipid structures of the cell membranes and the drug [11].

The literature review revealed that the reliability of these drugs which act as non-selective inhibitors of cyclooxygenase-1 and cyclooxygenase-2 isoenzymes are still under debate due to their potential and unpredictable gastrointestinal, hepatic, renal, hematological, and cardiac side effects [12–16].

The side effects of NSAID’S may also range from idiosyncratic skin eruptions to photosensitivity, ertyhema multiforme, leukocytoclastic vasculitis, and toxic epidermal necrolysis [17, 18]. NSAID’s were reported to exert adverse effects on patients with asthma, nasal polyps, and rhinitis [19]. These drugs were also found to be associated with tinnitus, hearing loss, coma, confusion, hallucination, depression, headache, syncope spells, dementia, personality changes, and cognitive dysfunction [20].

Attempts to repair the damaged tissues via biological methods were found to be intriguing by the orthopedic surgeons, as well as the researchers from other fields of medicine, and focus was given to conduct researches to protect or repair the damaged tissues, primarily the “joint cartilage” [1, 2].

Surprisingly, although it is evident that NSAID’s have both (i) a significant impact on a number of synovial mediators (e.g., cytokines) of a complex inflammatory process which have the potential to modify the chondroblastic and chondroclastic activity and (ii) an ability to further impair cartilage viability by decreasing the pH values in the inflammation site, to the best of our knowledge, no research investigating the possible chondrotoxic potentials of these organic acid-based drugs at the molecular level was conducted so far [1–5, 20–23].

The joint cartilage is devoid of neural, vascular, and lymphatic structures and nourished by passive and active transport processes. Since the outer portion of the synovial tissue is richer in terms of vascular supply, the nutrients first diffuse from the synovial tissue to the synovial fluid by diffusion. Then, the nutrients are transferred to the chondrocytes by either a second diffusion process that takes place through the pores within the cartilage or an active transport process in which the intermittently loaded pumps are used [24, 25]. Although the diffusion rates of NSAID’s are slower than other molecules and the concentrations of these drugs in the synovial fluids are rather stable, even short acting NSAID’s were reported to accumulate in the synovial fluid in concentrations slightly lower than their plasma concentrations [26].

In this research, the impact of flurbiprofen, a phenyl alkanoic derivative and the most frequently used NSAID, on the proliferation and differentiation of the human primary chondrocyte cultures were assessed in vitro using molecular biology techniques.

## Methods

### Materials

Collagenase type II enzyme (1 mg/ml) was purchased from Invitrogen Corporation. Hank’s balanced salt solution (HBSS)-1× (Cat ≠ 14025), Gibco penicillin-streptomycin, fetal calf serum, and Dulbecco’s modified Eagle’s medium (1000 mg glucose/L) were obtained from Sigma Chemical, St. Louis, USA. Sodium dodecyl sulfate (SDS) (Cat ≠ L4522), insulin-transferrin-selenous acid premix, and RPMI-1640 were obtained from Sigma-Aldrich Gmbh (Germany), and flurbiprofen (Majezik 100 mg) was supplied by Sanovel Corporation, Turkey.

A 3-(4,5-dimethylthiazol-2-yl)-2,5-diphenyltetrazolium bromide (MTT) commercial kit, (Vybrant MTT cell Proliferation assay (Cat ≠ V-13154), was purchased from Cell Biolabs, United States of America (USA). Stage-specific embryonic antigen-1 (SSEA-1; Human Mesenchymal Stem Cell Characterization Kit (Cat ≠ K36094-21A) was supplied from Celprogen Stem Cells Research and Therapeutics, CA, USA.

A laminar current cabinet (Cat ≠ NF–800 R) and incubator (Cat ≠ 06750) were supplied by the Nuve Company in Ankara, Turkey. Inverted light microscope (Cat ≠ CKX41, Olympus, USA) was used to monitor cell cultures. The images of cultures were obtained and processed using the Olympus cell soft imaging system program. MTT analysis and SSEA-1 protein expression values were determined using an ELISA reader (Mindray MR 96 A, PRC). Electron micrographs of cell cultures were obtained with an environmental scanning electron microscope (ESEM) (Quanta 250 FEG, Fei Company, Hillsboro, Oregon, USA).

### Study design and eligibility criteria

The researchers were blinded to the active ingredients in the NSAID applied in the cultures. All experiments were conducted in triplicate.

No drug was administered to primary chondrocyte cultures in group 1, and this group was used as the control group. The other groups were treated with 1 μM (group II), 10 μM (group III), 100 μM (group IV), 250 μM (group V), 500 μM (group VI), or 1000 μM (group VII) of flurbiprofen.

After 24, 48, and 72 h, the cell viability, toxicity, and proliferation, in addition to the expression levels of the SSEA-1 protein, which is a prechondrocytic precursor, were assessed using spectrophotometry. The surface morphology of the samples was simultaneously compared and evaluated using an inverted light microscope and ESEM.

Osteochondral tissues were obtained from patients (*n* = 17) with gonarthrosis who applied to the orthopedics and traumatology clinic and underwent total knee arthroplasty surgery. Of the 17 patients, tissues from those who had used NSAIDs (*n* = 5), disease-modifying antirheumatic drugs (DMARDs) (*n* = 4), and biological agents (*n* = 2) in the last month were excluded. Thus, tissues from six cases were included in this study.

#### Isolation and cell culture of primer human chondrocytes and application of the flurbiprofen to chondrocyte cultures

**Fig. 1:**
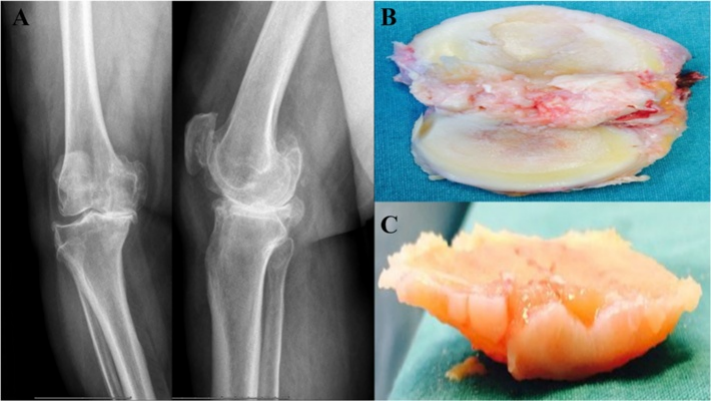
**a**–**c** Osteochondral tissue obtained during total knee replacement surgery. **a** Kellgren-Lawrence radiological scale grade IV. **b** Osteochondral tissue samples. **c** Chondral tissue samples

Osteochondral tissues from the proximal ends of the tibia and femur distal of the patients were resected during the total knee arthroplasty surgery (Fig. 1). These tissues were placed in a medium and transferred to the laboratory in aseptic conditions. The chondral tissues were separated from osteochondral tissues. The tissue samples were digested mechanically by a rongeur and enzymatically with collagenase type II enzyme, solubilized in HBSS and incubated at a humidified, 37 °C, 5 % CO_2_ incubator for 16 h. After incubation, digested tissue was centrifuged at 1000 rpm for 3 min and transferred to culture flasks to obtain chondrocyte primary cultures. Viable and attached cells were stained by trypan blue and counted. 1.5 × 10^4^ well/cell were placed in 96-well plates (for ELISA), 3.3 × 10^4^ well/cell were placed in 24-well plates (for SSEA-1), and 4.4 × 10^6^ cells were placed in a 10-mm petri dish for further experiments (for ESEM and invert microscopy) [1, 2, 27–30].

The flurbiprofen solutions were delivered in lightproof bottles to the researchers to do the analyses following letter coding. The researchers were blinded to the cases and also to the administered drugs.

### Microscopic analyses

Micro images of the cell organization of the cartilaginous tissue were recorded using phase-contrast confocal microscopy at magnifications of ×4, ×10, ×20, and ×40 before and after drug administrations. The Olympus Imaging System program was used to process the images.

To shed light on the surface topography and compositions of the chondrocytes, ESEM analysis was performed. In addition to cell morphology, extracellular matrix and characteristic structures of cell micro environment were recorded with ESEM.

To perform ESEM, cacodylate and glutaraldehyde buffer was used for fixation. Cell culture medium was removed with a gun pipettor and chondrocytes incubated with 2.5 % cacodylate and glutaraldehyde buffer for 2 h at room temperature. Afterwards, the samples were washed three times in pure cacodylate tampon and then analyzed [1, 2, 27–30].

Field emission guns (FEG) ion pumps were used for high vacuum. The images were recorded at a pressure of 220–221 Pa in ESEM vacuum mode, at a magnification of ×5000, resolutsion depth (HFW) of 82.9 μm, operating voltage of 5.00 kV, and wavelength-dispersive (WD) of 7.7–10.7 mm.

#### Determination of cell viability, toxicity, proliferation, and chondrogenic differentiation using molecular methods

The viability tests were carried out using an MTT kit. With the activity of dehydrogenase enzymes of viable cell mitochondria, blue formazan crystals form from the tetrazolium ring [1, 2, 27–30].

The cell viability and toxicity were analyzed in all experimental groups at 0, 24, 48, and 72 h after the application of the drug. Cell culture medium was discarded and 100 μl of the MTT stock solution were added to all the wells. Incubation for 150 min was then carried out at 37 °C in a dark environment. Ten microliters of DMSO was added and the samples were then incubated for another 10 min at 37 °C. Afterwards, absorbance was recorded at 540 nm (optical density (OD)) wavelength.

The cells viability of the control group before the application of flurbiprofen was assumed to be 100 %. The cell viability of drug administered groups was recorded after 24, 48, and 72 h and compared with that of the control group.

During the differentiation of human mesenchymal stem cells containing embryonic stem cells, the SSEA-1 protein is upregulated, whereas it is downregulated in cells lacking differentiation. To determine whether SSEA-1 was increased or decreased in the cultured cells in the drug treatment groups, a prechondrocytic human characterization ELISA kit was used [31]. The analyses were performed at 540 nm absorbance after 24, 48, and 72 h, using an ELISA reader.

### Statistical analysis

Descriptive statistics are shown as the mean ± standard deviation. In the analyses of the obtained data, results were evaluated by cell number, MTT cell viability, toxicity, and proliferation, and SSEA-1 protein expression. The Minitab R16 program was used in the statistical evaluation. All the evaluations were made at the 95 % confidence interval.

An analysis of variance (ANOVA) was conducted to detect significant differences across the groups. In the presence of differences across the groups, Tukey’s honest significant difference (HSD) post hoc multiple pairwise comparisons test was used to determine the difference and investigate the false positive, so the different averages across the experiment groups were evaluated.

Pearson’s correlation test was used to determine whether there was a direct relation between SSEA-1 expression and cell proliferation in the different subgroups. Tukey’s test resulted in a yes/no response to the hypothesis (e.g., are there significant differences between the wells, with *p* < 0.05). *P* < 0.01 was considered to be highly significant.

## Results

### Evaluation of microscopic analyses

**Fig. 2:**
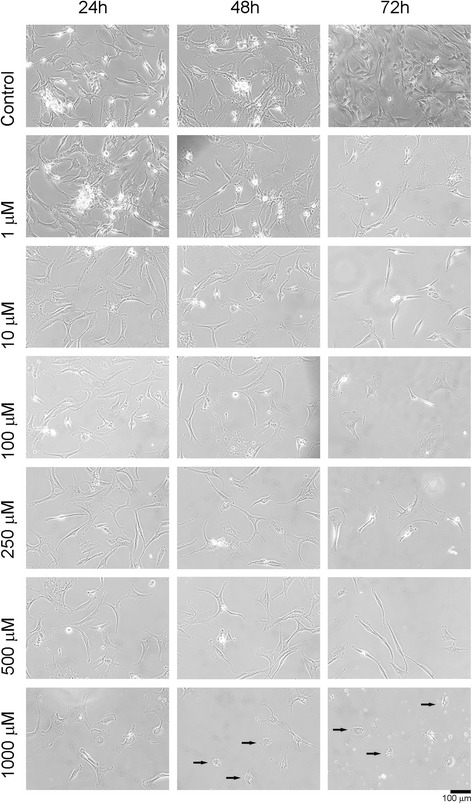
Inverted light microscopy image, showing the round shape of the cells caused by cytotoxicity (*black arrows*)

Cell viability and cell morphology were assessed by inverted light microscopy. Different doses of flurbiprofen (1–1000 μM) were applied to the cells for 24, 48, and 72 h, and microscopic changes were then recorded (Fig. 2).

As the dose of cytotoxic agent increased, the cell morphology changed, with the cells losing their specific shapes and becoming round shaped. Higher doses exerted greater effects on cell morphology than longer durations of lower doses. The changes were microscopically verified by ESEM and the MTT analyses.

**Fig. 3:**
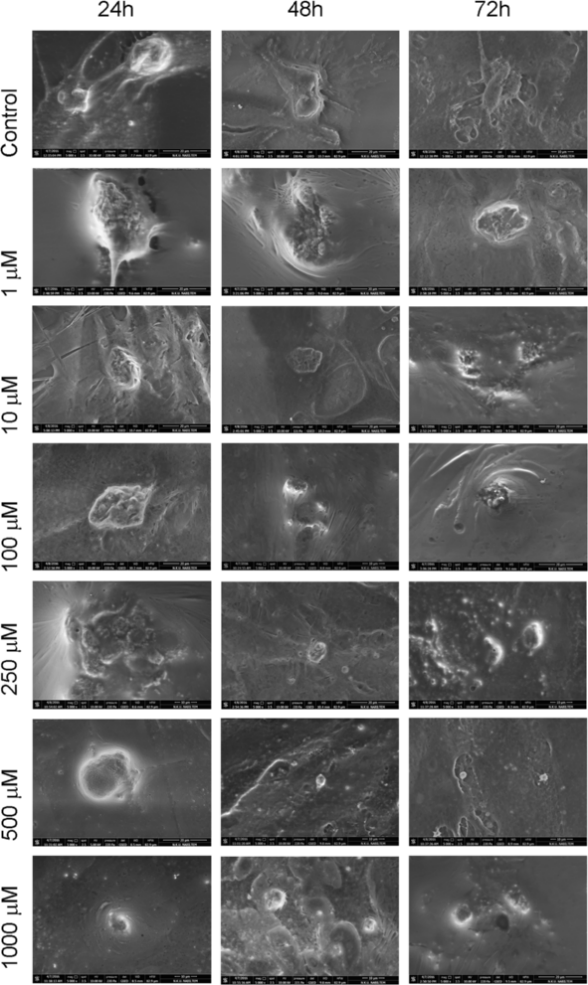
ESEM image of the surface morphology of the chondrocytes

In healthy chondrocytes showing chondroblastic activity, the surface properties of the cells in the images of the control group appeared normal. Viable cells were observed 24, 48, and 72 h after the application of flurbiprofen, but the viable cell concentration decreased with ascending doses. As shown by the ESEM analysis, deterioration of cell surface morphologies and loss of the extracellular matrix were evident in chondrocyte cultures after the application of 1000 μM of flurbiprofen (Fig. 3).

In all cases, dead cells were scattered throughout the culture, with decreased numbers of dead and living cells. In addition, the cell morphologies were altered, and the cells appeared to be contracted and to have lost their specific morphologies.

### Statistical evaluation of MTT and SSEA-1 protein expression

**Fig. 4:**
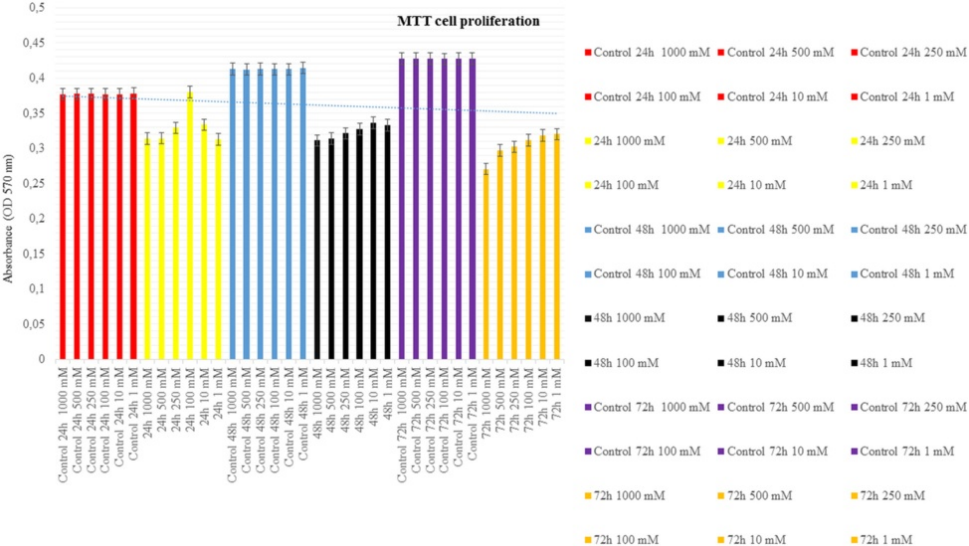
Change in the cell viability, toxicity, and proliferation

**Fig. 5:**
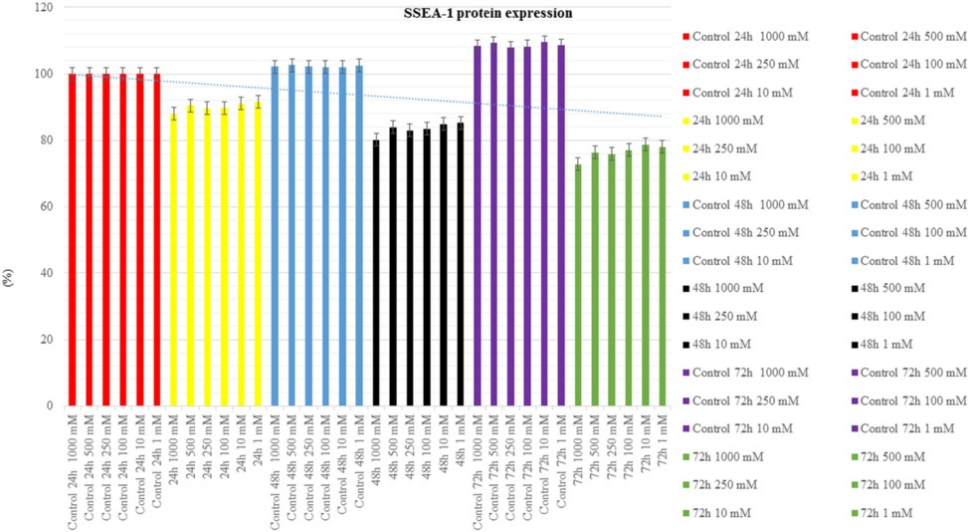
Change in the expression of SSEA-1

Cell viability, proliferation, and SSEA-1 protein expression values were lowest in the groups which flurbiprofen was administered 72 h at 1000 mM dose. The lowest toxicity rate was found in the group which 1 mM flurbiprofen was administered at 24 h. These results were statistically significant (*p* < 0.01) (*p* < 0.01; Figs. 4 and 5, Table 1).

**Table 1:** Statistical analyses; pairwise comparisons for SSEA-1 protein and MTT cell proliferation (grouping information using the Tukey method and 95 % confidence interval)

Hour	MTT cell viability, toxicity and proliferation^a^ (grouping^b^)
24	0.354158 (B)
48	0.368317 (A)
72	0.365417 (A)
Control	0.405917 (A)
Flurbiprofen	0.319344 (B)
	Stage-specific embryonic antigen-1
24	0.461242 (A)
48	0.450700 (A)
72	0.450008 (A)
Control	0.503494 (A)
Flurbiprofen	0.404472 (B)

^a^Variance of Analysis

^b^Tukey’s HSD post hoc multiple pairwise comparisons test

In the control groups which no agent was administered, cell viability, proliferation, and expression of SSEA-1 protein were found to be increased in time. In the experimental groups, viability, proliferation, and SSEA-1 protein expression were found to be decreased in comparison to the control groups. It was statistically confirmed that the time of exposure to the drug was a determinant in toxic effect (*p* = 0.037).

## Discussion

In all fields of medicine, ongoing efforts are being made to preserve the integrity of the healthy tissues or repair them in case of an injury using either biological or surgical methods. Moreover, the possible toxic effects of many frequently used drugs on the healthy or damaged tissues are also being investigated.

Joint cartilage is usually destructed secondary to degenerative disorders like osteoarthritis which are usually associated with the interactions of traumatic, mechanic, genetic, metabolic and biochemical factors and senility. Similarly, cutting, or crushing the cartilage tissue within the nasal septum during septoplasty or submucous resection operations were also reported to cause damage in a part of the cartilage tissue [32]. In addition to these factors, pharmaceutical preparations prescribed for the patients for the treatment of various disorders were also found to be responsible for the cartilage injury observed in these populations. The importance of keeping the integrity of the cartilage tissue was emphasized by many authors in several studies [1–3, 22, 28–31, 33, 34].

Therefore, the authors of the present study aimed to investigate the toxicity potential of a drug, flurbiprofen, which is frequently prescribed by not only orthopaedists and otorhinolaryngologists [8] but also other specialists, on the human primary chondrocyte cultures at the molecular level, in vitro.

As well as the structures within the joint cartilage is concerned, the most vulnerable structure following injury was reported to be the chondrocytes [33]. It was also reported that the majority of the drugs administered via different methods were found to accumulate in synovial fluids in significant concentrations [22, 34–36]. The cartilage tissue, however, is devoid of vascular, lymphatic, or neural structures. Therefore, the cartilage is nourished by either a perichondrium layer or a synovial fluid bathing the joint surface. Nutrients are reached to the chondrocytes through a double diffusion process. The nutrients (or drugs) first diffuse from the synovial tissue to the synovial fluid. Following this, they pass through the pores (6–8 nm in diameter) within the structure of the cartilage and reach the chondrocytes where a second diffusion takes place. In addition, a pumping system, composed of active transport and intermittent loading mechanisms, also have a significant contribution to the nourishment or transport of molecules to the chondrocytes.

The majority of drugs are composed of an organic acid backbone and they have a high affinity to bind plasma proteins. The pH value in the inflamed tissues is lower than other parts of the body and the acidic milieu enables rapid permeation of the plasma proteins into the inflamed tissues. Hence, plasma protein-bound NSAID’s readily accumulate at the sites of inflammation. Another factor regarding the pharmacological property of NSAID’S is their behavior in an acidic environment. As the pH decreases, the concentration of lipid soluble and non-ionized portion of the drug increases. Hence, the interaction of the lipid structures of the cell membranes is extended both in terms of duration and concentration [11]. Finally, the cartilage tissue has a basic environment and presence of an acidic synovial fluid in the vicinity would be detrimental for the chondrocytes.

When limited number of studies in which cartilage tissues or the chondrocyte directed toxicity was analyzed, it was found that they were carried out on animal models. However, the reliability of the results of these studies were questioned because the physiological responses and/or sensitivity of the tissues may be different in human subjects. Instead of animal models, other similar researches was found to use commercial cell lines, which are known to lose some genotypic and phenotypic features that they had in human bodies [1–3, 29, 30].

For this very reason, we did not use commercial cell lines. In this study, we used cartilage tissues harvested from patients during a surgical operation. During the application of knee prosthesis, primer chondrocyte cultures were prepared from cells obtained from undamaged chondral tissues of the resected articular surface.

Thanks to the primary cultures composed of chondral tissue obtained from the lateral compartment of the human joint cartilage, in addition to the chondrocytes, extensions like extracellular matrix could also be analyzed.

To the best of our knowledge, the present study is the first research that was conducted to assess the possible chondrotoxic potential of a phenyl alkanoic acid derivative, flurbiprofen, on the primary chondrocyte cultures of human, in vitro. For the first time, the structural changes in the chondrocytes in response to long-term exposure to the flurbiprofen, including loss of the integrity of the extracellular matrix, detachment of the cells from the surface of the cell-culture plates, and deformation of the specific cell morphology to attain a rather round appearance, could be observed in an in vitro setting via invert light microscopy and environmental scanning electron microscopy analysis (Figs. 2 and 3).

We did not only assess the viability, toxicity and proliferation by analyzing mitochondrial activity at the molecular level, but also assessed the level of chondrogenic activity, which is used for determining the prechondrocytic precursors, by measuring the extent of SSEA-1 protein expression.

In all groups exposed to varying doses of flurbiprofen, viability, proliferation, and SSEA-1 protein expression were found to be decreased in comparison to the control group. Duration of exposure was also proved to be a statistically significant factor in determining the degree of toxicity (*p* = 0.037 and *p* < 0.01, respectively).

We are of the opinion that the data obtained from our preliminary research may offer a new insight to the future researches regarding this topic. When prescribing such type of drugs for their analgesic and anti-inflammatory actions, their negative effects on the chondrocyte viability and the integrity of the extracellular matrix, in the long term, should be considered.

The present study has well based and strong in vitro results. Its major limitation is that it does not sufficiently reflect clinical setting. For this purpose, studies that evaluate clinical outcomes in vivo are needed.

## Conclusions

Phenyl alkanoic acid derivatives should not be used unnecessarily and be prescribed only for definitive indications. The treatment should be completed within the possible shortest time while the possible lowest dose is administered under strict surveillance.

## Abbreviations

DMARDs, disease-modifying antirheumatic drugs; ESEM, environmental scanning electron microscope; FEG, field emission guns; HBSS, Hank’s balanced salt solution; HSD, honest significant difference; MTT, 3-(4,5-dimethylthiazol-2-yl)-2,5-diphenyltetrazolium bromide; NSAIDs, non-steroidal anti-inflammatory drugs; OD, optical density; SDS, sodium dodecyl sulfate; SSEA-1, stage-specific embryonic antigen-1.7; WD, wavelength-dispersive
